# Practice makes perfect: self-reported adherence a positive marker of inhaler technique maintenance

**DOI:** 10.1038/s41533-017-0031-0

**Published:** 2017-04-24

**Authors:** Elizabeth Azzi, Pamela Srour, Carol Armour, Cynthia Rand, Sinthia Bosnic-Anticevich

**Affiliations:** 1 Woolcock Institute of Medical Research, University Of Sydney, Sydney, NSW Australia; 2 Sydney Local Health District, Sydney, NSW Australia; 3 Department of Medicine, John Hopkins University, Baltimore, MD USA

## Abstract

Poor inhaler technique and non-adherence to treatment are major problems in the management of asthma. Patients can be taught how to achieve good inhaler technique, however maintenance remains problematic, with 50% of patients unable to demonstrate correct technique. The aim of this study was to determine the clinical, patient-related and/or device-related factors that predict inhaler technique maintenance. Data from a quality-controlled longitudinal community care dataset was utilized. 238 patients using preventer medications where included. Data consisted of patient demographics, clinical data, medication-related factors and patient-reported outcomes. Mixed effects logistic regression was used to identify predictors of inhaler technique maintenance at 1 month. The variables found to be independently associated with inhaler technique maintenance using logistic regression (*Χ* ^2^ (3,*n* = 238) = 33.24, *p* < 0.000) were inhaler technique at Visit 1 (OR 7.1), device type (metered dose inhaler and dry powder inhalers) (OR 2.2) and self-reported adherent behavior in the prior 7 days (OR 1.3). This research is the first to unequivocally establish a predictive relationship between inhaler technique maintenance and actual patient adherence, reinforcing the notion that inhaler technique maintenance is more than just a physical skill. Inhaler technique maintenance has an underlying behavioral component, which future studies need to investigate.

## Asthma: Behavioral element to correct long-term inhaler techniques

Patients who consciously make an effort to perfect asthma inhaler technique will maintain their skills long-term. Elizabeth Azzi at the University of Sydney, Australia, and co-workers further add evidence that there is a strong behavioral component to patients retaining correct inhaler technique over time. Poor inhaler technique can limit asthma control, affecting quality of life and increasing the chances of severe exacerbations. Azzi’s team followed 238 patients to determine the key predictors of inhaler technique maintenance from factors including age, asthma knowledge and perceived future risks. Correct inhaler technique at initial assessment was the strongest predictor of long-term success, but this was strengthened further when patients reported good adherence to their own medication regimen. This suggests that maintaining correct inhaler technique is more than just a physical skill. Careful guidance towards this ‘practice makes perfect’ approach may improve patients’ long-term technique maintenance.

## Introduction

Poor inhaler technique and non-adherence to treatment are major problems in the management of asthma.^1–3^ Between 72–83% (depending on device) of people with asthma are not using their inhalers correctly^4^ and up to 90% of patients are not taking them regularly as prescribed.^5^ This has significant consequences, with incorrect technique being associated with sub-optimal dosing of prescribed medication, resulting in reduced response to treatment, poor asthma control^6–9^ and non-adherence to medication, which can lead to unstable asthma symptoms, significant morbidity, and premature death.^10, 11^ Both poor inhaler technique and non-adherence continue to be problematic.^12^

There is much literature that has helped our understanding of non-adherence to asthma medications as a health behavior. Non-adherence to asthma medication is linked to many factors, and can be organized into five interacting dimensions: social-economic, condition-related, therapy-related, healthcare system-related, and patient-related factors.^13^ Research has shown that adherence to asthma medications can be improved with complex interventions that target patients’ perceptions of their asthma, their beliefs about asthma medication, motivation, self-efficacy, forgetfulness, beliefs, attitudes, understanding, and inhaler technique.^14^

With regards to inhaler technique, our understanding is somewhat different. While there is substantial research highlighting the importance and effectiveness of delivering regular education on inhaler technique,^9, 12, 15–18^ maintaining technique over time remains problematic.^19^ Fifty percent of patients who are taught how to use their inhalers correctly subsequently experience difficulty in maintaining correct technique, irrespective of device type or duration of follow-up after education,^19, 20^ little is known about ‘why’ patients do not maintain correct technique.

While inhaler technique has traditionally been viewed as a skill/dexterity-based acquisition, the research of Ovchinikova et al. (2010) suggests that inhaler technique maintenance may be related to patient psychosocial factors. In an exploration of predictors of inhaler technique maintenance, it was found that the type of inhaler device, asthma control, and motivation were related to inhaler technique maintenance. This research was the first to identify a relationship between inhaler technique and individual psychosocial factors, and further challenges the paradigm that repeated instruction and education is not the only key to inhaler technique maintenance. In fact, Ovichinikova et al., (2010) did not find an association between past technique education and inhaler technique maintenance.

This research raises questions about the concept of inhaler technique and its relationship to other health behaviors, including adherence to medications.^12, 21^ Understanding how psychosocial factors influence inhaler technique maintenance is therefore the next critical step to determining effective solutions to poor inhaler technique. The aim of this study is to gain a deeper understanding of the way in which asthma management practices, health behaviors and psychosocial factors relate to inhaler technique maintenance in a cohort of people with asthma. It is hypothesized that there may be key disease-related, patient-related or behavior-related factors that predict inhaler technique maintenance. Health-care professionals (HCP) could use these to identify patients at risk of long-term incorrect inhaler use.

## Results

**Fig. 1:**
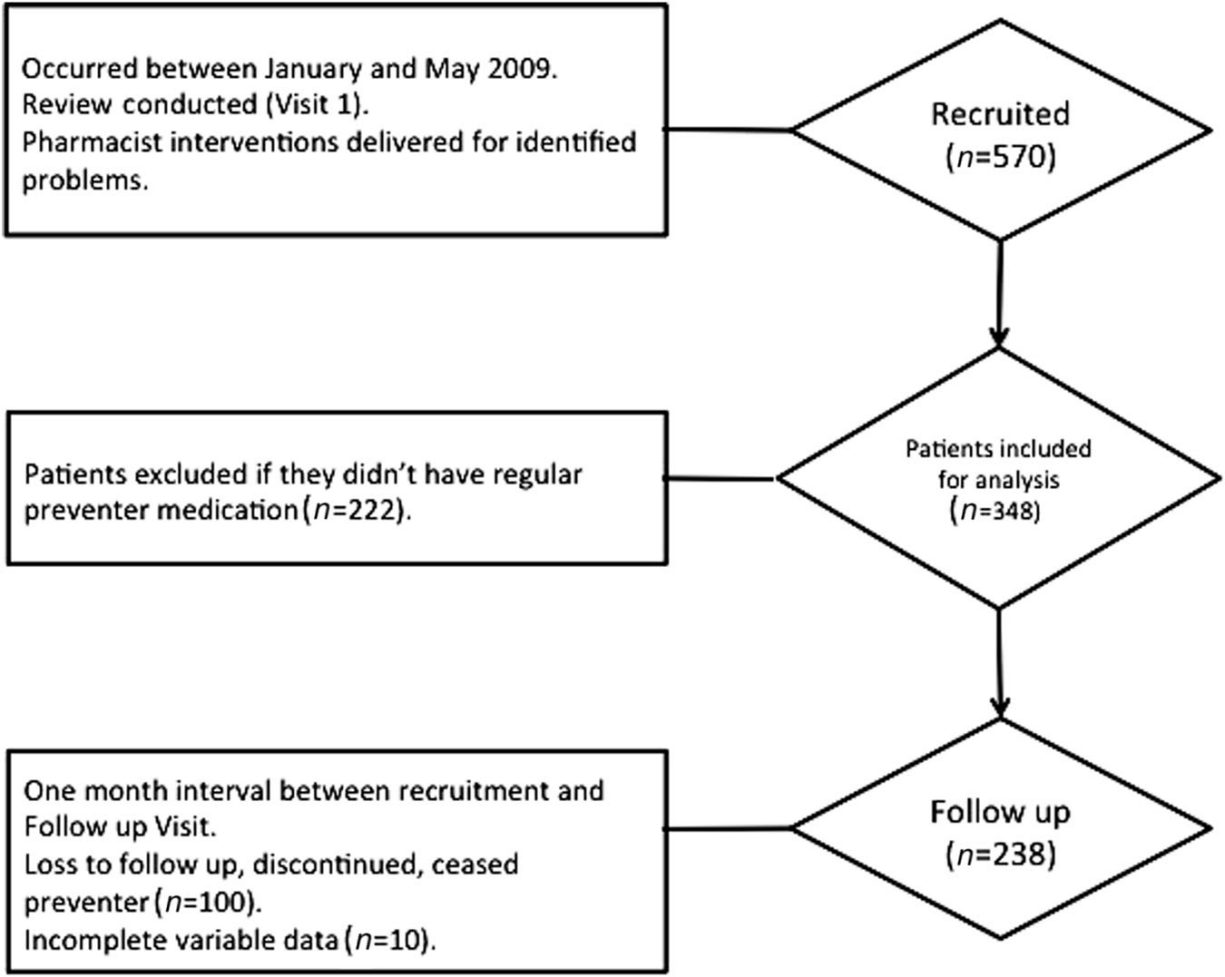
Consort diagram of patients recruited and completing the study

In total, 96 pharmacies collected data from 570 patients who fulfilled the inclusion criteria at Visit 1. Of these, 348 patients were taking preventer/combination medication on a regular basis and from these, Visit 2 data was available for 238 patients (Fig. 1).

### Demographics

The mean age of patients was 52.5 (±16.3 (standard deviation (S.D.)); 150 (63%) were female and 78% of patients (*n* = 186/238) reported an asthma-related comorbidity (allergic rhinitis, hay fever or eczema).

### Asthma history, asthma control, perceived control, asthma quality of life

Twenty percent of patients (*n* = 48/238) reported a hospital admission or emergency room presentation due to asthma in the past year; 16% (*n* = 38/238) reported that they have had a life threatening attack in the last 5 years.

Seventy five percent (*n* = 179/238) of patients reported that they had received information from a health professional in regards to asthma. Fifty percent (*n* = 118/238) reported to have had their asthma reviewed by a general practitioner or specialist in the last 6 months; 21% (*n* = 51/238) owned an asthma action plan, of which 86% (*n* = 44/51) reported that they knew how to use their asthma action plan.

Asthma control was determined using the Symptom and Activity Tool^22^ and 75% (*n* = 179/238) of patients were classified as having poor asthma control, 21% (*n* = 51/238) had fair asthma control and only 3% (*n* = 7/238) had good asthma control. Mean perceived control of asthma score was 24.8 ± 5.2 (±S.D.), ranging from 12–42 with the lower score indicating better-perceived asthma control.

Asthma quality of life scores^23^ were calculated on a 10-point scale, the mean total asthma quality of life score was 4.3 ± 1.5 (±S.D.), ranging from 2–9.5 with the lower score indicating less impact of asthma on quality of life.

Asthma knowledge questionnaire^24^ consisted of 12 true or false questions. The mean score was 7.5 ± 2.53 (±S.D.), ranging from 0–12; a higher score indicating better asthma knowledge.

### Future risk of medication non-adherence

Future risk of medication non-adherence was evaluated using the Brief Medication Questionnaire. The mean total Brief Medication Questionnaire score was 2.95 ± 1.95 (±S.D.). In evaluating the validated subscales, mean values of 1.4 ± 1.30 (±S.D.), 0.5 ± 0.69 (±S.D.), and 1.01 ± 0.66 (±.) for the Regimen Screen, Beliefs Screen, and Recall Screen, respectively.^25^ Sixty-eight percent (*n* = 162/238) of patients scored ≥1 in the Brief Medication Questionnaire Regimen Screen indicating a future risk of medication non-adherence.^25^

### Inhaler technique

Thirty percent (*n* = 71) of patients utilized a pressurized metered dose inhaler (pMDI), 38% (*n* = 90) a Turbuhaler® (TH), and 32% (*n* = 77) an Accuhaler® (ACC). The proportion of patients demonstrating correct technique throughout the study according to inhaler device type is summarized in Table 1. There were no statistically significant differences between the patients who maintained vs. those who did not maintain correct technique with respect to the use of different types of dry powder inhalers (DPI), the TH or ACC (*p* *>* .05).

**Table 1:** Proportion of patients with correct inhaler technique per device

	Visit 1^a^	Visit 2
Correct technique (*n* = 238)	24% (56/238)	50% (118/238)
Correct technique per device		
pMDI technique (*n* = 71/238)	25% (18/71)	40% (28/71)
DPI technique (*n* = 167/238)	23% (38/167)	54% (90/167)
TH technique (*n* = 90/167)	17% (15/90)	52% (47/90)
ACC Technique (*n* = 77/167)	30% (23/77)	56% (43/77)

^a^ All patients who demonstrated incorrect technique at Visit 1 were trained to mastery

### Determinants of inhaler technique maintenance

The independent variables that showed significant bivariate associations with ‘correct inhaler technique maintenance’ were inhaler technique at Visit 1 (correct 24% vs. incorrect 76%), preventer type (DPI 54% vs. MDI 40%) and Brief Medication Questionnaire Regimen subscale (*n* = 238, *p* < 0.05) (Table 2).

**Table 2:** Independent variables with significant bivariate association with ‘correct inhaler technique maintenance’

Variable/characteristic	Maintained	Did not maintain	Pearson co-efficient	*p* value
Inhaler technique at Visit 1				
Correct inhaler technique (*n* = 56)	44	12	24.622	0
Incorrect inhaler technique (*n* = 182)	74	108		
Regimen Score				
Adherent (scores <1)	47	29	12.557	0.028
Future risk of non-adherence (scores ≥1)	71	91		
Device type DPI/MDI				
DPI	90	77	4.164	0.041
MDI	28^a^	43^b^		

^a^ 9 patients using a MDI with a spacer maintained correct technique

^b^ 6 patients using a MDI with a spacer did not maintain correct technique

Age, gender, asthma control, asthma knowledge, asthma quality of life, perceived control of asthma and the total future risk of non-adherence as measured by the total Brief Medication Questionnaire score as well as the Belief and Recall subscales were not associated with inhaler technique maintenance.

Based on the association analysis, inhaler technique at Visit 1, preventer type and future risk of medication non-adherence as it’s related to Regimen screen (self-reported 7-day adherence) were the only three independent variables subsequently included for analysis in the mixed effects logistic regression analysis.

Mixed effects logistic regression modeling (allowing for clustering by introducing pharmacy identifiers (ID) as a random effect) determined that inhaler technique at Visit 1, device type and Regimen screen (self-reported adherent behavior in the prior 7 days) were predictors of inhaler technique maintenance ( *Χ* ^2^ (3,*n* = 238) = 33.24, *p* < 0.000 (Omnibus test of Model Coefficients)) (Table 3). The model correctly classified 80.3% of cases and explained between 13.0 and 17.6% of the variance in inhaler technique maintenance.

**Table 3:** Logistic regression model variables showing significant predictive likelihood for correct inhaler technique maintenance

Predictor	Coding	B	SE	Deviance	df	*p* value	Odds ratio	95% CI for odds ratio	
								Lower	Upper
Inhaler technique at VISIT 1	0 = Correct technique (*n* = 56)	1.965	.455	17.431	1	.000	7.131	3.104	18.922
	1 = Incorrect technique (*n* = 182)								
Regimen Score	Score range 1–7	.275	.127	4.498	1	.031	1.317	1.027	1.701
Device type	0 = DPI (*n* = 167)	.791	.355	5.344	1	.026	2.205	1.116	4.554
	1 = pMDI (*n* = 71)^a^								

Outcome variable coding for inhaler technique maintenance: 0 = maintained correct inhaler technique, 1 = did not maintain correct inhaler technique.

^a^ MDI and MDI with a spacer groups were combined under pMDI to achieve sufficient group size for logistic regression analysis

The strongest predictor of inhaler technique maintenance was correct inhaler technique at Visit 1, followed by device type (DPI patients being more likely to maintain correct technique) and Regimen Screen Score (with patients reporting better 7 day adherence more likely to maintain correct technique), controlling for all other factors in the model.

The tests of assumptions in this study were not violated, and a sufficient sample size was obtained for each of three independent predictors, with 79.33 cases per variable. There were no significant interactions and no collinearity problem with any variable (all tolerance values >0.88).

## Discussion

### Main findings

This study explored a wide range of clinical and patient-related factors in an attempt to identify predictors of inhaler technique maintenance. While age, asthma control, perceived asthma control, asthma quality of life and asthma knowledge were not associated with inhaler technique maintenance, it was determined that individuals who had correct inhaler technique at the initial assessment, used a DPI (TH or ACC) and had self-reported adherent behavior in the prior 7 days were more likely to maintain correct inhaler technique over time.

### Interpretation of findings in relation to previously published work

The strongest predictor of inhaler technique maintenance was inhaler technique mastery at initial assessment. This finding is consistent with previous research^26^ and was expected, i.e., individuals who are able to use their inhaler correctly are more likely to do so over time. It could be assumed that this is a result of patients having had effective and repeated training in the use of their inhalers.^12^ However, this study also found that correct technique does not guarantee maintained mastery. We found that approximately one-fifth of patients who were assessed as having device mastery initially, were not able to demonstrate maintenance of that mastery at follow-up. This finding questions the ‘stability’ of ‘correct’ inhaler technique, and the need to continue to evaluate inhaler technique over time. The asthma guidelines state that inhaler technique needs to be rechecked and education reinforced on a regular basis, due to the instability of inhaler technique.^16, 18^

Type of inhaler device used was another predictor of inhaler technique maintenance. Patients using DPIs (TH or ACC) were more likely to maintain correct technique compared to those using pMDIs. This finding is consistent with previous research^26^ and not surprising given the added complexity required to use a pMDI, in particular the need for inhalation upon actuation,^27^ which is not required for the use of DPIs.^28^

The final predictor of inhaler technique maintenance identified was self-reported adherent behavior in the 7 days prior to initial inhaler technique assessment, as evaluated with the Regimen Screen subscale of the Brief Medication Questionnaire.^25^ The Brief Medication Questionnaire is a validated instrument used to predict future risk of non-adherence.^25^ The Brief Medication Questionnaire groups the study population into repeat, sporadic and no non-adherence and is constructed from three subscales based on three facets of non-adherence: Regimen Screen (five-items related to self-reported recent adherence), Beliefs Screen (two-items related to patient attitudes to medication use) and Recall Screen (two-items related to forgetfulness).^25^ This is the first study to determine an association between a measure of adherence and inhaler technique maintenance. That is, patients who self-reported better adherent behavior in the week prior to initial assessment were more likely to maintain correct technique, while patients who reported non-adherent behavior in the week prior to initial assessment were less likely to maintain correct technique, regardless of their asthma knowledge/beliefs, asthma control, perceived asthma control or asthma quality of life. This particular finding has important conceptual/theoretical and practical implications.

In this research, while overall future risk of medication non-adherence wasn’t associated with inhaler technique maintenance, it was only the Regimen Screen that was identified as a predictor of inhaler technique maintenance. The Regimen Screen subscale is associated with the self-reported adherence behavior over the preceding 7 days i.e., actual patient adherence. Regimen screen has a 95% accuracy, 80% sensitivity, 100% positive predictive value, and specificity for detecting repeat non-adherence.^25^ Due to the self-reported nature of this questionnaire, 7 days is used within the questionnaire as a shorter recall period and to reduce the potential of self-reporting error; both over and under estimating adherence.^25^ The results of this study suggest that patients who are non-adherent to their asthma medications are at a greater risk of not maintaining correct inhaler technique. Therefore, poor inhaler technique maintenance is likely a reflection of continued non-adherence or perhaps a consequence.

In past studies inhaler technique and adherence have been considered as separate constructs. While incorrect inhaler technique has been noted as unintentional non-adherence by Jimmy et al. (2011),^29^ ‘inhaler technique’ has traditionally been considered a physical skill and therefore has not strictly fulfilled the definition of ‘adherence’. Adherence is defined as ‘the degree to which patient behaviors coincide with the clinical recommendation of healthcare providers’^30^ and is considered to be intrinsically linked to patient behavior and patient motivation.^10^ Underpinning the constructs of motivation to adhere to medications and behaviors are patient perceptions, patient beliefs and attitudes about illness, treatment and interactions with their HCP.^10, 13, 16^ HCP patient interactions cannot be discounted in a clinical setting, as HCPs are responsible for training patients to use an inhaler and provide information to improve adherence through motivation and change in behavior.^13^ Unfortunately, research indicates that HCPs are not providing patients with adequate information regarding adherence and proper inhaler technique, with a large proportion of HCPs not being able to use inhalation devices themselves.^31, 32^ The potentially negative impact of this on patient motivation to master, maintain correct technique and adhere to prescribed inhalation therapy can not be ignored. Due to the complexity of ‘adherence’, different types of non-adherence exist and in this study an attempt was made to identify any potential relationship between the different types of adherence and inhaler technique maintenance.

### Implications for future research, policy and practice

This research draws our attention to several aspects in more depth for future research. Firstly, this research challenges our understanding of the relationship between adherence and inhaler technique maintenance. Therefore, future research needs to explore this relationship in greater depth to determine/confirm the way in which adherence may be able to predict inhaler technique issues over time. Secondly, future research needs to explore the nature of errors made, various inhaler device types, the ‘stability’ of inhaler technique and the ‘stability/consistency’ in the way in which it is assessed. Understanding the implications of these findings is critical to clinical practice and the tailoring of education to better support the complexity required in using different devices over time. In considering all of this, future research needs to consider the reliability and validity of the measure of adherence used.

It is also important to consider the implications of this research on health care delivery and practice. Practice guidelines promote the need to assess and educate patients with regards to their use of inhalers,^18^ but in practice, this does not happen.^12^ Therefore, by identifying those individuals who are likely to have problems in maintaining correct technique, even following education, HCPs can make better decisions as to who requires follow-up with regards to this particular aspect of asthma medication management. This research indicates that there is a way to identify patients at risk of poor inhaler technique through an evaluation of recent adherence and the device that they are using. Further, this research uncovers a novel opportunity whereby HCPs may be able to address the issue of non-adherence and inhaler technique maintenance simultaneously under the more socially desirable guise of ‘quality use of respiratory medicines’.

### Strengths and limitations of this study

In considering the results of this study it is also important to consider the study cohort and the generalizability of these findings. Recent Australian data identified that 46% of participants have poorly controlled asthma and only 32% of patients were using their preventer medication on a regular basis.^1^ Patients recruited for this study come from a real-life cohort who had established asthma and were included based on criteria that identified patients at risk of poorly controlled asthma. Therefore it is not surprising that 76% of patients in this study had poor asthma control. Therefore, the strength of this study lies in the fact that it focuses on patients at risk in a real-life scenario. A limitation of this study is that the findings of this research may not be able to be generalised to all people with asthma (i.e., individuals who are not prescribed preventer therapy or are significantly younger than the study cohort).^1^ Future research is needed to investigate adherence and inhaler technique maintenance in younger patients, and patients with newly diagnosed asthma, who are perhaps more influential in terms of disease beliefs and adherence behaviours.

### Conclusion

In conclusion, while most HCP can acknowledge that adherence and inhaler technique maintenance are important aspects of medication use this study goes beyond this and establishes a relationship between inhaler technique maintenance, which has been traditionally considered a physical skill and adherence, a concept traditionally limited to health beliefs a cognition. Highlighting the need to consider inhaler technique maintenance beyond a physical skill. The results reinforce the notion that ‘practice makes perfect’. Implying that optimal adherence to asthma medication provides patients with a variety of opportunities to reinforce and practice correct inhaler technique, thereby retaining the technique over time. Further research is needed to elucidate the conceptual relationship between these two domains in order to identify opportunities for HCPs to address the issues of poor inhaler technique and adherence more effectively.

## Methods

Ethical approval from the University of Sydney Human Research Ethics committee was obtained prior to commencement of the study; all participants provided written informed consent.

### Study design

This study utilized data from a quality-controlled longitudinal community pharmacy dataset (containing de-identified data related to asthma reviews conducted by community pharmacists over a 12-month period). The data originated from 96 community pharmacies across Australia. These pharmacies where allocated individual pharmacy identification numbers (pharmacy ID) to ensure anonymity. Patients included in the data set underwent extensive asthma reviews delivered by trained pharmacists.^4^ Individuals in the data set fulfilled the following inclusion criteria: people with asthma who were aged ≥18 years and fulfilling ≥1 criterion from the modified Jones Morbidity Index.^33^ Individuals were excluded if they had a terminal illness, did not speak English, were enrolled in another study, or did not self-administer their medicines/inhalers. The data consisted of clinical data easily measureable in community pharmacy as well as additional data related to more advanced clinical, patient-reported outcomes (see below). As the focus of this research was inhaler technique maintenance, the data relating to a review visit (Visit 1 during which pharmacists reviewed the patient’s asthma status and delivered appropriate interventions) and the first follow-up visit i.e., the 1-month follow-up visit (Visit 2) were analyzed.

### Data collection

Data relating to patient demographics, clinical and patient-reported outcomes were collected as part of the asthma review (Visit 1). This included data relating to smoking status, current medications and ownership of written action plans.

Specific clinical data collected included history of asthma and asthma control (Asthma Control Symptom and Activity Tool,^22^ validated against the Asthma Control Questionnaire for use in community pharmacy)^34, 35^.

Patient-reported outcomes relating to asthma quality of life,^23^ the patient’s perception of their extent to which their asthma was well controlled,^36^ asthma knowledge,^24^ future risk of medication non-adherence and potential barriers were reviewed using the Brief Medication Questionnaire^25^ and its validated subscales of Regimen Screen (self-reported recent (7-day) adherence), Belief Screen and Recall Screen.^25^

Medication-related factors such as inhaler technique (based on previously used inhaler technique checklists)^4^ were assessed. Inhaler technique and future risk of medication non-adherence were evaluated only for medications recommended for regular use i.e., preventer medications (inhaled corticosteroids) and combination medications (inhaled corticosteroids and long-acting β2-agonist).^16^ Patients who maintained correct inhaler technique at Visit 2 i.e., were able to complete all steps of the inhaler technique checklist correctly, were labeled as “maintained correct inhaler technique”. Patients who did not complete all steps of the inhaler technique checklist correctly at Visit 2 were labeled as “did not maintain correct inhaler technique”. The types of delivery devices used to deliver the preventer/combination medication was recorded and reclassified, as either dry powder inhaler (DPI) (TH and ACC) or pMDI.

Following the collection of data at Visit 1, pharmacists addressed any management needs identified, including inhaler technique training and followed-up patients in 4 weeks’ time for Visit 2 data collection.

Table 4 summarizes the data collected at Visit 1 and Visit 2. Supplementary table 1 provides further detail relating to these outcomes and the tools used.

**Table 4:** Data measures and collection points

	Measure	Visit 1	Visit 2
Clinical			
	Asthma control	✓	✓
	Medication profile	✓	✓
	Future risk of medication non-adherence	✓	
	Inhaler technique	✓	✓
	Asthma knowledge	✓	
	Hospital emergency department visits and admissions	✓	
Patient-related			
	Asthma quality of Life	✓	
	Asthma perceived control	✓	

### Statistical analysis

Data analysis was conducted using SPSS version 23^TM^ (SPSS-IBM, Chicago, IL, USA) and Program R (R Core Team, 2016).^37–39^ Descriptive analysis was performed on demographic data, asthma history and asthma control.

To test for potential predictors of inhaler technique maintenance, preliminary analysis by Pearson’s *χ* ^2^ was used to test for differences in categorical variables or Mann–Whitney *U* tests for continuous variables. Inhaler technique maintenance was dichotomized into “maintained correct inhaler technique” and “did not maintain correct inhaler technique” as the dependent variable. The independent variables: asthma control, perceived control of asthma, asthma quality of life (and its subscales), inhaler device type, correct or incorrect inhaler technique at Visit 1, and future risk of medication non-adherence (Brief Medication Questionnaire and its calculated subscales: Regimen Screen, Belief Screen and Recall Screen), were statistically examined for suitability for inclusion in the logistic regression modeling by examining the presence of any binary associations between inhaler technique maintenance and each independent variable.

Due to the exploratory nature of this study, no prior assumptions of relationships between factors were made, therefore variables for inclusion in the analysis were selected based on the above-outlined statistical approach.^40^ To account for any cluster effect (i.e., correlations of patients within pharmacies),^41^ a mixed effects logistic regression was performed including pharmacy ID as a random effect. Prior to execution of the mixed effects logistic regression modeling (accounting for clustering),^37–39^ testing for underlying assumptions were carried out.^41^

## Electronic supplementary material

Supplementary Table 1
