# Supplementary material for: Practice makes perfect: self-reported adherence a positive marker of inhaler technique maintenance
Source: NPJ Prim Care Respir Med. 2017 Apr 24;27:29. doi: 10.1038/s41533-017-0031-0 (PMC5435088; doi:10.1038/s41533-017-0031-0)
Supplement: Supplementary file 1 — Supplementary Table 1 [file 41533_2017_31_MOESM1_ESM.docx]

APPENDIX

**Supplementary Table 1:** Tools/instruments use to evaluate and record review data.

| Review data | Tools/instruments | Data Management |
| --- | --- | --- |
| Asthma Control | Asthma Control Symptom and Activity Tool [22] | Asthma Control Symptom and Activity Tool which classifies asthma control as “good”, “fair”, and “poor” [22]. |
| Asthma Quality of Life | Impact of Asthma on Quality of Life Questionnaire (IAQLQ) [23] | A 20-item questionnaire with subscales for breathlessness, mood disturbance, social disruption and concerns for health. Total scores and subscores were scaled 0–10, where a higher score indicates a greater impact of asthma on quality of life [23]. |
| Perceptions of asthma control | Perceived Control of Asthma Questionnaire (PCAQ) [36] | An 11-item questionnaire, total score range 11-55, with lower scores representing perceptions of better ability and confidence to manage and control asthma [36] |
| Future risk of non-adherence | Brief Medication Questionnaire [25] | A sum of 3 subscales (Regimen, belief and recall). A higher value indicates a higher risk of the potential of non-adherence [25]. |
| Barriers to non-adherence | i. Belief Screen of the Brief Medication Questionnaire [25]  ii. Recall Screen of the Brief Medication Questionnaire [25] | 2-item Belief Screen that identifies patient’s perceptions of drug effects and medication concerns. Score of > 1 indicates positive screen for belief barriers [25].  2-item Recall Screen relating to potential memory difficulties experienced by the patient. Score of >1 indicates positive screen for recall barriers [25]. |
| Medication Adherence | ii. Self-reported 7 day medication adherence: Regimen Screen of the Brief Medication Questionnaire [25] | 5-items relating to patient self-report of how they took each medication in the past week. Score of >1 indicates positive screen for recall barriers. |
| Inhaler technique | Device-specific inhaler technique checklists [4] | Each patient was given a score for inhaler technique for each device that they used. The score for each device was then converted to a percentage/proportion i.e. the percentage of steps performed correctly out of the maximum number of steps required for the use of each device. This allowed comparisons between different devices with different numbers of steps required for administration. |
| Asthma Knowledge | Consumer Asthma Knowledge Questionnaire (CQ) [24] | A 12-item questionnaire comprising of a series of true/false questions. A higher score indicates better asthma knowledge. |
